# A New Lignanamide from the Root of *Lycium yunnanense* Kuang and Its Antioxidant Activity

**DOI:** 10.3390/molecules23040770

**Published:** 2018-03-27

**Authors:** Xin-Heng Zheng, Yuan-Peng Huang, Qiu-Ping Liang, Wei Xu, Ting Lan, Guang-Xiong Zhou

**Affiliations:** Guangdong Province Key Laboratory of Pharmacodynamic Constituents of Traditional Chinese Medicine and New Drugs Research, Institute of Traditional Chinese Medicine and Natural Product, College of Pharmacy, Jinan University, Guangzhou 510632, China; 13751866247@163.com (X.-H.Z.); pennhuang@sina.com (Y.-P.H.); desert_liang@163.com (Q.-P.L.); xwnail2003@163.com (W.X.); lanting842358215@163.com (T.L.)

**Keywords:** *Lycium yunnanense* Kuang, lyciumamide K, ECD calculations, antioxidant activities, oxygen radical absorbance capacity (ORAC) assay

## Abstract

A new lignanamide (**1**), lyciumamide K, together with four known analogues (**2**–**5**), was isolated from the root of *Lycium yunnanense* Kuang. Based on HR-ESI-MS, NMR spectral data and quantum chemistry ECD calculations, the structure of this new compound was confirmed, including its absolute configuration. Evaluation of the antioxidant activity of compounds **1**–**5** in the oxygen radical absorption capacity (ORAC) assay showed that they all exhibited significant antioxidant activities. Particularly, compound **1** showed the best activity with ORAC values (U/mol) of 7.90 ± 0.52. Thus, the new lignanamide may be a good source of bioavtive and protective compounds.

## 1. Introduction

**Figure 1:**
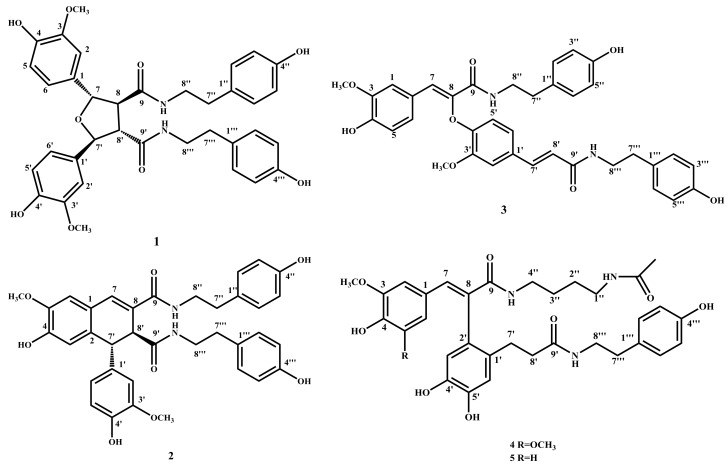
The structures of compounds **1**–**5**.

There are 80 species of *Lycium* (Solanaceae) throughout the world, mainly in South Africa, Asia, temperate Europe and South America. China has seven species and three varieties, which are mainly distributed in the northwest and north of China [1]. Cortex Lycii, that is the root bark of *Lycium barbarum* L., has been widely used in traditional Chinese medicine treatment of night sweats, pneumonia, cough and diabetes, etc. [2]. Several types of compounds have been isolated from these plants, including lignanamides, alkaloids, flavonoids, cyclic peptides, lignans, terpenes and phenolic glycosides [3,4,5,6,7,8,9]. Meanwhile, the antioxidant properties of the fruits of *Lycium barbarum* L. have been intensively studied for years [10,11,12]. The literature has demonstrated that polysaccharides, carotenoids, and especially phenolic compounds (flavonoids and phenolic acids) obtained from the fruits of *L. barbarum* performed effective antioxidant activities [13]. *Lycium yunnanense* Kuang is another species of plant in the same genus, and is commonly found in such places as Luquan, Jingdong, Funing, Yanshan and Malipo in Yunnan province, China, born in the wet sandy soil of riversides or jungles in elevation of 700~2200 m. In order to further study the active ingredients of the related traditional Chinese medicine, a systematic chemical study was conducted on the ethyl acetate fraction of the ethanol extract of the root of *Lycium yunnanense* Kuang. As a result, a new lignanamide (**1**) as well as four known compounds (**2**–**5**) were isolated (see Figure 1). Here, we elaborate the isolation and structural analysis of this lignadamide and its antioxidant activity.

## 2. Results and Explanation

### 2.1. Structural Feature of the Compound **1**

**Figure 2:**
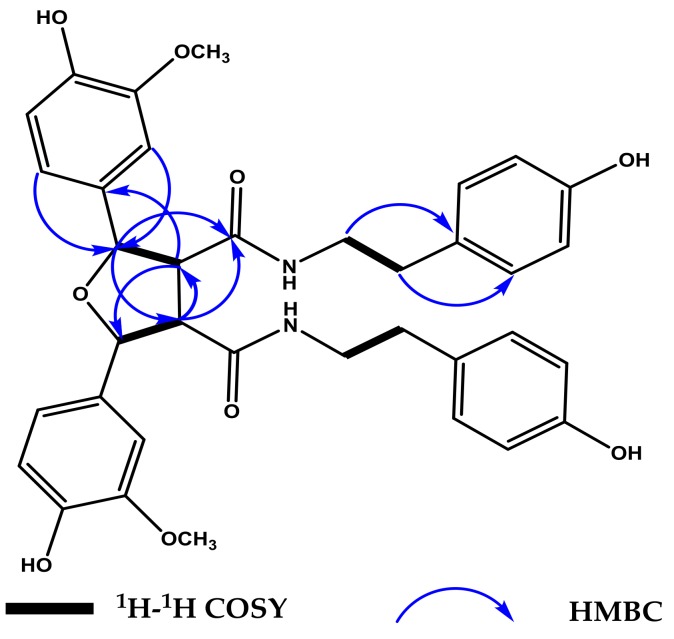
Key ^1^H–^1^H COSY and HMBC correlations for compound **1**.

Compound **1** was obtained as white amorphous powder. The molecular formula of **1** was determined to be C_36_H_38_N_2_O_9_ on the basis of the sodiated molecular ion peak observed at *m*/*z* 665.2474 [M + Na]^+^ by HR-ESI-MS, which indicated 19 degrees of unsaturation. The ^1^H, ^13^C-NMR and HSQC data of **1** (Table 1, see Supplementary Materials) suggested the presence of tyramine moiety (δ_H_ 6.84 (2H, d, *J* = 8.4 Hz), 6.64 (2H, d, *J* = 8.4 Hz), 3.39 (1H, t, *J* = 7.2 Hz), 3.17 (1H, t, *J* = 7.2 Hz), 2.53 (1H, t, *J* = 7.2 Hz), 2.59 (1H, t, *J* = 7.2 Hz) and δ_C_ 156.8, 131.0, 130.7 × 2, 116.2 × 2, 42.5, 35.7), a 1,3,4-trisubstituted aromatic ring [δ_H_ 7.03 (1H, d, *J* = 1.5 Hz), 6.79 (1H, d, *J* = 8.1 Hz), 6.81 (1H, dd, *J* = 1.5, 8.1 Hz), and δ_C_ 149.3, 147.8, 133.2, 120.5, 116.2, 110.8], and a carbonyl group (δ_C_ 172.1). Compound **1** presented characteristic ^1^H-NMR and ^13^C-NMR signals for tetrahydrofuran-type lignans with the chemical shifts of two methines (δ_H_ 3.34 (1H, dd, *J* = 2.8, 6.4 Hz), δ_C_ 60.6) and two benzylic methines (δ_H_ 5.26 (1H, d, *J* = 2.8 Hz), δ_C_ 86.2) substituted by oxygen. These data indicated the presence of oxygen-bearing carbons, which were further confirmed by DEPT and HSQC data [14]. All the ^1^H and ^13^C-NMR data only accounted for half the number of atoms expected for its molecular formula (C_36_H_38_N_2_O_9_), suggesting that **1** is a symmetrical dimer of two completely identical moieties (monomer units). The interpretation of the NMR data of **1** revealed that the monomer part was structurally similar to the known compound *N*-*trans*-feruloyltyramine [15], which co-occurs with **1** in the same extract. The ^13^C-NMR chemical shifts and quaternary status of C-8/C-8′ (δ_C_ 60.6), C-7/C-7′ (δ_C_ 86.2) suggested that the two monomer units were connected via tetrahydrofuran (THF) unit [14]. This was further confirmed by the HMBC correlations from H-2 to C-7, from H-6 to C-7, and from H-7 to C-2, C-6, C-8/C-8′ and C-9, and from H-8 to C-1, C-7/C-7′, C-8′ and C-9, (see Figure 2). 

The relative configuration of Compound **1** was confirmed by multiple data. From the NOESY spectrum, there was NOE correlations between H-6 and H-7, and between H-2 and H-8 in methanol-*d*_4_. Moreover, the coupling constant values of both H-7 with H-8, and H-7′ with H-8′ were 2.8 Hz, and the coupling constant value of H-8 with H-8′ was 6.4 Hz. Finally, we calculated the configurations between C-7/C-7′, C-8/C-8′ with the lowest energy of compound **1** in ChemDraw [16]. All of these results suggested that the substitution of the THF ring of **1** should possess a 7, 8-*trans*/8, 8′-*trans*/7′, 8′-*trans* relative configuration [17,18,19].

**Figure 3:**
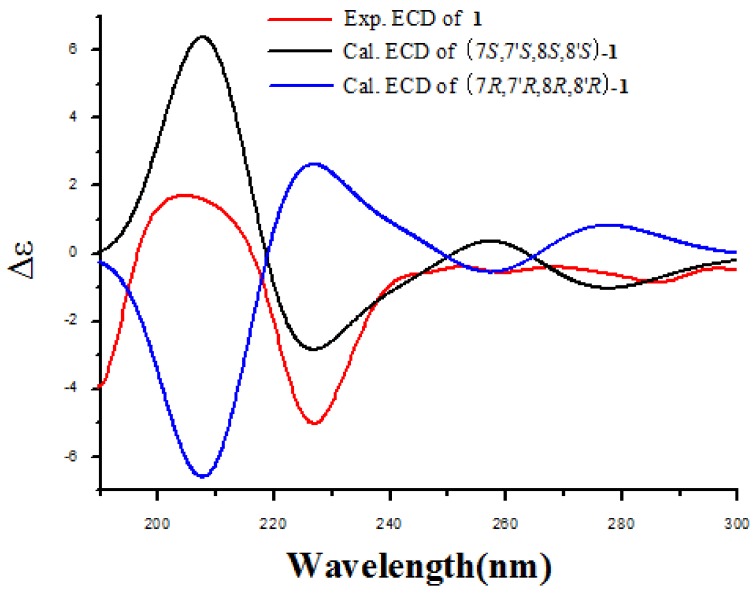
Experimental ECD spectra of compound **1** and the calculated ECD spectra of (7*S*,7′*S*,8*S*,8′*S*)-**1** and (7*R*,7′*R*,8*R*,8′*R*)-**1**.

**Figure 4:**
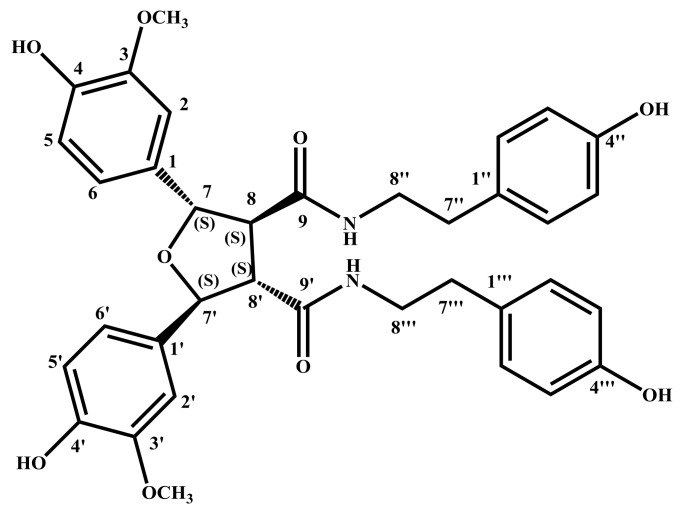
The chemical structure of **1**, indicated the absolute configurations of chiral centers.

The absolute configuration of **1** was determined by ECD calculation. In the UV spectrum, the maximum absorption around 205 nm exhibited the same UV features for all the lignanamides. Following a standard procedure (SYBYL version 2.0) for the prediction of ECD spectra, the conformational ensemble of **1** in solution was investigated by a molecular mechanics conformational search via the MMFF94 force field. Finally, the lowest energy conformers of (7*S*,7′*S*,8*S*,8′*S*)-**1** and (7*R*,7′*R*,8*R*,8′*R*)-**1** were obtained. Subsequently, the pairs of isomers were re-optimized using DFT at the B3LYP/6-31+G (d) level in gas phase by the GAUSSIAN 09 program with the measured CD data. By comparing the experimental ECD spectrum with the calculated ECD spectra, the absolute configuration of **1** was determined to be (7*S*,7′*S*,8*S*,8′*S*)-**1** (Figure 3). According to these data, the structure of **1** was completely determined (Figure 4), and named lyciumamide K.

Compounds **2**–**5** were identified as known compounds cannabisin D (**2**), cannabisin F (**3**), (*E*)-2-(4,5-dihydroxy-2-{3-[(4-hydroxyphenethyl)amino]-3-oxopropyl}phenyl)-3-(4-hydroxy-3,5 -dimethoxyphenyl)-*N*-(4-hydroxyphenethyl)acrylamide (**4**), (*E*)-2-(4,5-dihydroxy-2-{3-[(4-hydroxy phenethyl)amino]-3-oxopropyl}phenyl)-3-(4-hydroxy-3-methoxyphenyl)-*N*-(4-acetamidobutyl) acrylamide) (**5**) on the basis of their NMR and MS data referring to the literature [5,20,21].

### 2.2. Antioxidant Activity

**Figure 5:**
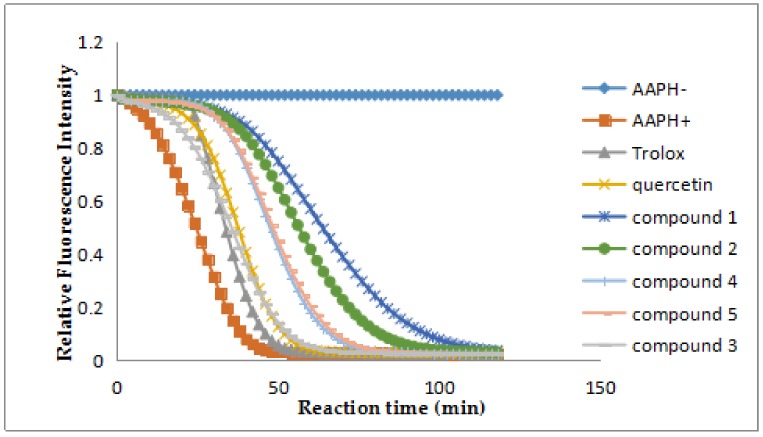
Fluorescence decay curves induced by AAPH in the presence of compounds **1**–**5** at 1 μM.

The isolated compounds **1**–**5** exhibited obvious antioxidant activities by the ORAC assay with quercetin as the positive control. According to the result of ORAC assay, compounds **1**, **2**, **4** and **5** showed strong activities with ORAC values (U/mol) of 7.90 ± 0.52, 6.44 ± 0.48, 4.60 ± 0.30, 4.80 ± 0.25, respectively (Table 2, Figure 5). In particular, Compound **1** is the best antioxidant among them.

## 3. Materials and Methods

### 3.1. General Experimental Procedures

IR data were recorded with a Nicolet Impact 410-FTIR instrument (Thermo, San Jose, CA, USA). Optical rotations were conducted with JASCO digital polarimeter (JASCO Corporation, Tokyo, Japan). UV spectra were measured on a JASCO V-550 UV/VIS spectrometer (JASCO Corporation, Tokyo, Japan). HR-ESI-MS were obtained from an Agilent 6210 LC/MS TOF mass spectrometer (Agilent Technologies, Santa Clara, CA, USA.). NMR spectra were carried out on a Bruker AV-300 and AV-600 spectrometer (Bruker Instrument, Inc., Zurich, Switzerland). HPLC was performed on an Agilent 1200 HPLC system equipped with a diode array detector, using a column A (Ultimate XB-C18, 5 μm, 4.6. 250 mm, Welch, Potamac, MA, USA) for analysis and a semi-preparative HPLC column B (Ultimate XB-C18, 5 μm, 10 × 250 mm, Welch, Potamac, MA, U.S.A.) for purification. Sephadex LH-20 (25–100 mm) was purchased from Pharmacia and open column chromatography (CC) was conducted on silica gel (200–300 mesh, Haiyang Chemical Group Corporation, Qingdao, China). HSGF254 silica gel TLC plates (0.2 mm thickness, 200 × 200 mm, Qingdao Marine Chemical, Qingdao, China) were used for routine analysis and chemical analysis. The spraying reagent used for TLC detection was 10% H_2_SO_4_ in EtOH. 2,2′-azobis (2-methylpropionamidine) dihydrochloride (AAPH), Fluorescein sodium salt and 6-hydroxy-2,5,7,8-tetramethylchroman-2-carboxylic acid (Trolox) were purchased from Macklin (Shanghai, China). All the other reagents and solvents were provided by Tianjin Damao Chemical Company (Tianjin, China).

### 3.2. Plant Material

The root of *Lycium yunnanense* Kuang was collected from Malipo County, Wenshan Prefecture, Yunnan Province in June 2015. Its original plant was identified as the solanaceous plant *Lycium yunnanense* Kuang by Dr. En-De Liu at Kunming Institute of Botany, Chinese Academy of Sciences.

### 3.3. Extraction and Isolation

The root of *Lycium yunnanense* Kuang (2.75 kg) was smashed into powder, then extracted twice times with 95% EtOH (10 L) and once time with 80% EtOH (10 L). The solvent of the combined extracts was evaporated and the residue (276 g) was suspended in H_2_O and successively partitioned with petroleum ether and EtOAc. The EtOAc fraction (40 g) was separated to a silica gel column, a stepwise gradient elution of CH_2_Cl_2_/MeOH [100:1, 50:1, 30:1, 20:1, 15:1, 10:1, 5:1 and 2:1 (*v*/*v*)] was used and 12 fractions (Fr 1-12) were got through TLC analysis. Fraction 7 (5.9 g) was subjected to a silica gel column eluting with a step-gradient of CH_2_Cl_2_/MeOH (from 20:1 to 2:1) to afford 5 subfractions Fr 7-1–Fr 7-5. Fr 7-1 (1.2 g) was chromatographed over a Sephadex LH-20 column (100% MeOH) and was further purified with semi-preparative HPLC (solvent system: MeOH/H_2_O (40:60)) to yield **1** (7.0 mg, t_R_ = 50.24 min) and **2** (4.0 mg, t_R_ = 42.0 min). Fr 7-2 (1.1 g) was further purified with semi-preparative HPLC (solvent system: MeOH/H_2_O (52:48)) to yield **3** (15.1 mg, t_R_ = 44.28 min). Fraction 9 (3.0 g) was chromatographed via ODS column at a proportion of MeOH/H_2_O [30–100%, *v*/*v*] to get 3 fractions Fr 9-1–Fr 9-3. Fr 9-2 (742 mg) was chromatographed over a Sephadex LH-20 column (100% MeOH) and was further purified with semi-preparative HPLC (solvent system: MeOH/H_2_O (38:62)) to yield **4** (5.5 mg, t_R_ = 26.25 min). Fr 9-3 (653 mg) was further purified with semi-preparative HPLC (solvent system: MeOH/H_2_O (35:65)) to yield **5** (26.7 mg, t_R_ = 42.0 min). All semi-preparative HPLC methods were operated in the flow rate of 3 mL min^−1^ and detected at 210 nm (UV).

**Lyciumamide K (1):** White amorphous powder; ${\left[\alpha \right]}_{D}^{20}$ + 29.4 (c 0.50, MeOH); UV (MeOH) *λ*_max_ (log ε): 205.2 (4.87), 228.3 (4.47) nm; IR (KBr) ν_max_: 3449, 1638 cm^−1^. CD (MeOH) *λ*_max_ (Δε): 227.2 (−4.52), 207.8 (+1.88) nm; ^1^H-NMR (CD_3_OD, 600 MHz) and ^13^C-NMR (CD_3_OD, 150 MHz) data, see Table 1; HR-ESI-MS (positive): *m*/*z* 665.2474 [M + Na]^+^ (calcd. for C_36_H_38_N_2_O_9_Na, 665.2470).

### 3.4. Computational Details for ECD of Compound **1**

In general, the systematic random conformational analysis of two possible stereoisomers ((7*S*,7′*S*,8*S*,8′*S*)-**1** and (7*R*,7′*R*,8*R*,8′*R*)-**1**) of **1** was carried out via random searching in the Sybyl-X 2.0 using the MMFF94S force field with an energy cutoff of 2.5 kcal mol^−1^. Subsequently, the conformers were further optimized using DFT at the B3LYP/6-31+G (d) level in gas phase by using Gaussian09 program (Gaussian, Inc., Wallingford, CT, USA). The energies, oscillator strengths, and rotational strengths (velocity) of the first 60 electronic excitations were calculated using the TD-DFT methodology at the B3LYP/6-311++G (d, p) level in vacuum. The ECD curves were simulated by the overlapping Gaussian function (half the bandwidth at 1/e peak height, σ = 0.2 eV). In order to get the final spectra, The ECD spectrum were weighted by the Boltzmann distribution of each conformer and their relative Gibbs free energy (ΔG). Theoretical ECD spectrum of the corresponding enantiomer was obtained by directly inverse of the ECD spectrum of the calculated model molecule, respectively. The calculated ECD spectrum of (7*S*,7′*S*,8*S*,8′*S*)-**1** and (7*R*,7′*R*,8*R*,8′*R*)-**1** were subsequently compared with the experimental spectra, respectively [22,23,24].

### 3.5. Antioxidant Activity

Oxygen Radical Absorbance Capacity (ORAC) Assay [25,26,27]. Briefly, 20 μL, 10 μM sample or 20 μL, 20 μM Trolox to be tested (prepared with 75 mmol/L potassium phosphate buffer, pH 7.4) and potassium phosphate buffer were added to each well of 96-well plate, respectively. Fluorescein sodium salt (final concentration of 63 nmol/L) and 140 μL AAPH (final concentration 12.8 mmol/L) were quickly added to the 96-well plate in a temperature of 37 °C in a fluorescence analyzer. Fluorescence was immediately read at 538 nm with 485 nm excitation wavelength. The decrease of fluorescence was read every two minutes for 2 h at 37 °C, with 5 s stirring before measurement. Trolox solution was used as the internal control. The area under the curve (AUC) was calculated as:

AUC = 2 × (f_0_ +f_1_ + … + f_n-1_ + f_n_) − f_0_ − f_n_ (1)

where f_0_ was the initial fluorescence read at 0 min, and f_n_ was the fluorescence read at corresponding time. The Net AUC were different values between the AUC of the blank and each sample. ORAC values were expressed as:

ORAC value = [(AUCSample − AUC + AAPH)/(AUCTrolox − AUC + AAPH)] × (molarity of Trolox/molarity of sample) = (Net AUC_Sample_/Net AUC_Trolox_) × (molarity of Trolox/molarity of sample) (2)

## 4. Conclusions

A new lignanamide dimer (**1**) as well as four known ones (**2**–**5**) were isolated from the root of *Lycium yunnanense* Kuang. The structure of the new lignanamide was elucidated on the basis of HR-ESI-MS, NMR spectral data and especially quantum chemistry ECD calculations. The antioxidant activities of all compounds were tested by ORAC assay and the results showed that all of them exhibited good antioxidant activities. These findings suggested the potential of these compounds as a medicinal supplement for the treatment and prevention of diseases related to oxidation stress. 

## Figures and Tables

**Table 1:** ^1^H and ^13^C-NMR data of compound **1** (CD_3_OD, δ in ppm, *J* in Hz).

NO.	^13^C-NMR (δ_C_^a^)	^1^H-NMR (δ_H_^b^)
1, 1′	133.2	-
2, 2′	110.8	7.03 (d, 1.5)
3, 3′	149.3	-
4, 4′	147.8	-
5, 5′	116.2	6.79 (d, 8.1)
6, 6′	120.5	6.81 (dd, 1.5, 8.1)
7, 7′	86.2	5.25 (d, 2.8), 5.26 (d, 2.8)
8, 8′	60.6	3.34 (dd, 2.8, 6.4)
9, 9′	172.1	-
1′′, 1′′′	131.0	-
2′′, 2′′′	130.7	6.84 (d, 8.4)
3′′, 3′′′	116.2	6.64 (d, 8.4)
4′′, 4′′′	156.8	-
5′′, 5′′′	116.2	6.64 (d, 8.4)
6′′, 6′′′	130.7	6.84 (d, 8.4)
7′′, 7′′′	35.7	2.53 (t, 7.2), 2.59 (t, 7.2)
8′′, 8′′′	42.5	3.17 (t, 7.2), 3.39 (t, 7.2)
3, 3′-OCH_3_	56.4	3.88 (s)

^a^ Measured at 150 MHz. ^b^ Measured at 600 MHz.

**Table 2:** Antioxidant activities of compounds **1**–**5**.

Compound	ORAC (U/mol) ^a^
**1**	7.90 ± 0.52
**2**	6.44 ± 0.48
**3**	2.16 ± 0.21
**4**	4.60 ± 0.30
**5**	4.80 ± 0.25
Quercetin ^b^	2.59 ± 0.21

Note: U was equivalent Trolox of 1 mol. ^a^ Mean ORAC values ± SD (*n* = 10) are shown. ^b^ Used as positive control.

## Supplementary Materials

The following are available online. IR, UV, CD, HR-ESI-MS, NMR spectra of compounds **1** as well as other supporting data.

## Abbreviations

HR-ESI-MS	High Resolution Electron Spray Ionization Mass
NMR	Nuclear Magnetic Resonance
ECD	Electronic Circular Dichroism
HPLC	High Performance Liquid Chromatography
^1^H-^1^H COSY	Two Dimensional ^1^H Correlation
DEPT	Distortionless Enhancement by Polarization Transfer
HSQC	^1^H-Detected Heteronuclear Single-Quantum Coherence
HMBC	^1^H-Detected Heteronuclear Multiple-Bond Correlation
NOESY	Nuclear Overhauser Effect Spectroscopy

## References

[1] Committee of National Pharmacopoeia (2015). China Pharmacopoeia.

[2] Xie L.W., Atanasov A.G., Guo D.A., Malainer C., Zhang J.X., Zehl M., Guan S.H., Heiss E.H., Urban E., Dirsch V.M. (2014). Activity-guided isolation of NF-κB inhibitors and PPARγ agonists from the root bark of *Lycium chinense* Miller. J. Ethnopharmacol..

[3] An Y.W., Zhan Z.L., Xie J., Yang Y.N., Jiang J.S., Feng Z.M., Ye F., Zhang P.C. (2016). Biocative octahydroxylated C_21_ steroids from the root bark of *Lycium chinense*. J. Nat. Prod..

[4] Yang Y.N., An Y.M., Zhan Z.L., Xie J., Jiang J.S., Feng Z.M., Ye F., Zhang P.C. (2017). Nine new compounds from the root bark of *Lycium chinense* and their α-glucosidase inhibitory activity. RSC Adv..

[5] Zhang J.X., Guan S.H., Feng R.H., Wang Y., Wu Z.Y., Zhang Y.B., Bi K.S., Guo D.A. (2013). Neolignanamides, Lignanamides, and other phenolic compounds from the root bark of *Lycium chinense*. J. Nat. Prod..

[6] Lee D.G., Park Y., Kim M.R., Jung H.J., Seu Y.B., Hahm K.S., Woo E.R. (2004). Anti-fungal effects of phenolic amides isolated from the root bark of *Lycium chinense*. Biotechnol. Lett..

[7] Funayama S., Zhang G.R., Nozoe S. (1995). Kukoamine B, a spermine alkaloid from *Lycium chinense*. Phytochemistry.

[8] Yahara S., Shigeyama C., Ura T., Wakamatsu K., Yasuhara T., Nohara T. (1993). Cyclic peptides, acyclic diterpene glycosides and other compounds from *Lycium chinense* Mill. Chem. Pharm. Bull..

[9] Yao X., Peng Y., Xu L.J., Li L., Wu Q.L., Xiao P.G. (2011). Phytochemical and biological studies of *Lycium* medicinal plants. Chem. Biodivers..

[10] Le L., Chiu F., Ng K. (2007). Identification and quantification of antioxidants in *Fructus lycii*. Food Chem..

[11] Chang R.C., So K.F. (2008). Use of anti-aging herbal medicine, *Lycium barbarum*, against aging-associated diseases. What do we know so far?. Cell. Mol. Neurobiol..

[12] Li X.M. (2007). Protective effect of *Lycium barbarum* polysaccharides on streptozotocin-induced oxidative stress in rats. Int. J. Biol. Macromol..

[13] Wang C.C., Chang S.C., Stephen L.B., Chen B.H. (2010). Isolation of carotenoids, flavonoids and polysaccharides from *Lycium barbarum* L. and evaluation of antioxidant activity. Food Chem..

[14] Henrici A.K., Kaloga M.K., Eich E.K. (1994). Jacpaniculines, the first lignanamide alkaloids from the convolvulaceae. Phytochemistry.

[15] Chen H., Li Y.J., Sun Y.J., Gong J.H., Du K., Zhang Y.L., Su C.F., Han Q.Q., Zheng X.K., Feng W.S. (2017). Lignanamides with potent antihyperlipidemic activities from the root bark of *Lycium chinense*. Fitoterapia.

[16] (2015). ChemDraw Software.

[17] Chaves M.H., Roque N.F. (1997). Amides and lignanamides from *Porcelia macrocarpa*. Phytochemistry.

[18] Matsushita H., Miyase T., Ueno A. (1991). Ligan and terpene glycosides from *Eplmedium sagittatum*. Phytochemistry.

[19] Fu S.N., Wang F., Li H.Y., Bao Y.X., Yang Y., Shen H.F., Lin B.R., Zhou G.X. (2016). Secondary metabolites from marine-derived *Streptomyces antibioticus* strain H74-21. Nat. Prod. Res..

[20] Sakakibara I., Ikeya Y., Hayashi K., Marrsuhashi H. (1992). Three phenyldihydronaphthalene lignanamides from fruits of *Cannabis sativa*. Phytochemistry.

[21] Sakakibara I., Ikeya Y., Hayashi K., Marrsuhashi H. (1995). Three acyclic *bis*-phenylpropane lignanamides from fruits of *Cannabis sativa*. Phytochemistry.

[22] (2013). Sybyl Software.

[23] Frisch M.J., Truck G.W., Schlegel H.B. (2009). Gaussian 09, Revision A.01.

[24] Stephens P.J., Harada N. (2010). ECD cotton effect approximated by the Gaussian curve and other methods. Chirality.

[25] Huang D., Ou B., Hampsch-Woodill M., Flanagan J.A., Prior R.L. (2002). High-throughput assay of oxygen radical absorbance capacity (ORAC) using a multichannel liquid handling system coupled with a microplate fluorescence reader in 96-well format. J. Agric. Food Chem..

[26] Xu J.K., Yao X.S., Hiroshi K. (2006). Oxygen radical absorbance capacity assay and its application. Chin. J. Pharmacol. Bull..

[27] Claudia G.I., Erika S., Nathalie B., Bruno B., Atikorn P., Maria C.F. (2016). Antioxidant activity of protocatechuates evaluated by DPPH, ORAC, and CAT methods. Food Chem..

