# Supplementary material for: A New Lignanamide from the Root of Lycium yunnanense Kuang and Its Antioxidant Activity
Source: Molecules. 2018 Mar 27;23(4):770. doi: 10.3390/molecules23040770 (PMC6017700; doi:10.3390/molecules23040770)
Supplement: Supplementary file 1 [file molecules-23-00770-s001.pdf]

Supplementary Materials

A New Lignanamide from the Root of *Lycium yunnanense* Kuang and Its Antioxidant Activity

Xin-Heng Zheng, Yuan-Peng Huang, Qiu-Ping Liang, Wei Xu, Ting Lan, Guang-Xiong Zhou *

Guangdong Province Key Laboratory of Pharmacodynamic Constituents of Traditional Chinese Medicine and New Drugs Research, Institute of Traditional Chinese Medicine and Natural Product, College of Pharmacy, Jinan University, Guangzhou 510632, China; 13751866247@163.com (X.-H.Z.); pennhuang@sina.com (Y.-P.H.); desert_liang@163.com (Q.-P.L.); xwnail2003@163.com (W.X.); lanting842358215@163.com (T.L.)

* Correspondence: guangxzh@sina.com; Tel.: +86-20-8522-1469; Fax: +86-20-8522-1559

Contents

Figure S1-1. ^{1}H NMR spectrum (600 MHz) of compound **1** in methanol-d_4.....4

Figure S1-2. ^{13}C NMR spectrum (150 MHz) of compound **1** in methanol-d_4....4

Figure S1-3. DEPT-135 spectrum (150 MHz) of compound **1** in methanol-d_4...5

Figure 1-4. ^{1}H-^{1}H COSY spectrum (600 MHz) of compound **1** in methanol-d_4 5

Figure S1-5. HMQC spectrum (150 MHz) of compound **1** in methanol-d_4 6

Figure S1-6. HMBC spectrum (150 MHz) of compound **1** in methanol-d_4........6

Figure S1-7. NOESY spectrum (600 MHz) of compound **1** in methanol-d_4 7

Figure S1-8. HR-ESI-MS of compound **1**...7

Figure 1-9. IR spectrum of compound **1**..8

Figure 1-10. CD spectrum and UV spectrum of compound **1** in MeOH 8

Figure S1-11. B3LYP/6-31 G optimized lowest energy 3D conformer of **1**.......9

Table S1. B3LYP-Calculated relative energies (kcal mol^{-1}) and conformational population (%) for the most stable conformers of (7*S*,7'*S*,8*S*,8'*S*)-**1**. 9

Figure S2-1. ^{1}H NMR spectrum (600 MHz) of compound **2** in methanol-d_4...10

Figure S2-2. ^{13}C NMR spectrum (150 MHz) of compound **2** in methanol-d_4..10

Figure S2-3. HR-ESI-MS of compound **2**...11

Figure S3-1. ^{1}H NMR spectrum (600 MHz) of compound **3** in methanol-d_4...11

Figure S3-2. ^{13}C NMR spectrum (150 MHz) of compound **3** in methanol-d_4..12

Figure 3-3. HR-ESI-MS of compound **3**..12

Figure S4-1. ^{1}H NMR spectrum (300 MHz) of compound **4** in methanol-d_4...13

Figure S4-2. ^{13}C NMR spectrum (75 MHz) of compound **4** in methanol-d_4....13

Figure S4-3. HR-ESI-MS of compound **4**...14

Figure S5-1. ^{1}H NMR spectrum (300 MHz) of compound **5** in methanol-d_4 ...14

Figure S5-2. ^{13}C NMR spectrum (75 MHz) of compound **5** in methanol-d_4 15

Figure S5-3. HR-ESI-MS of compound **5** 15

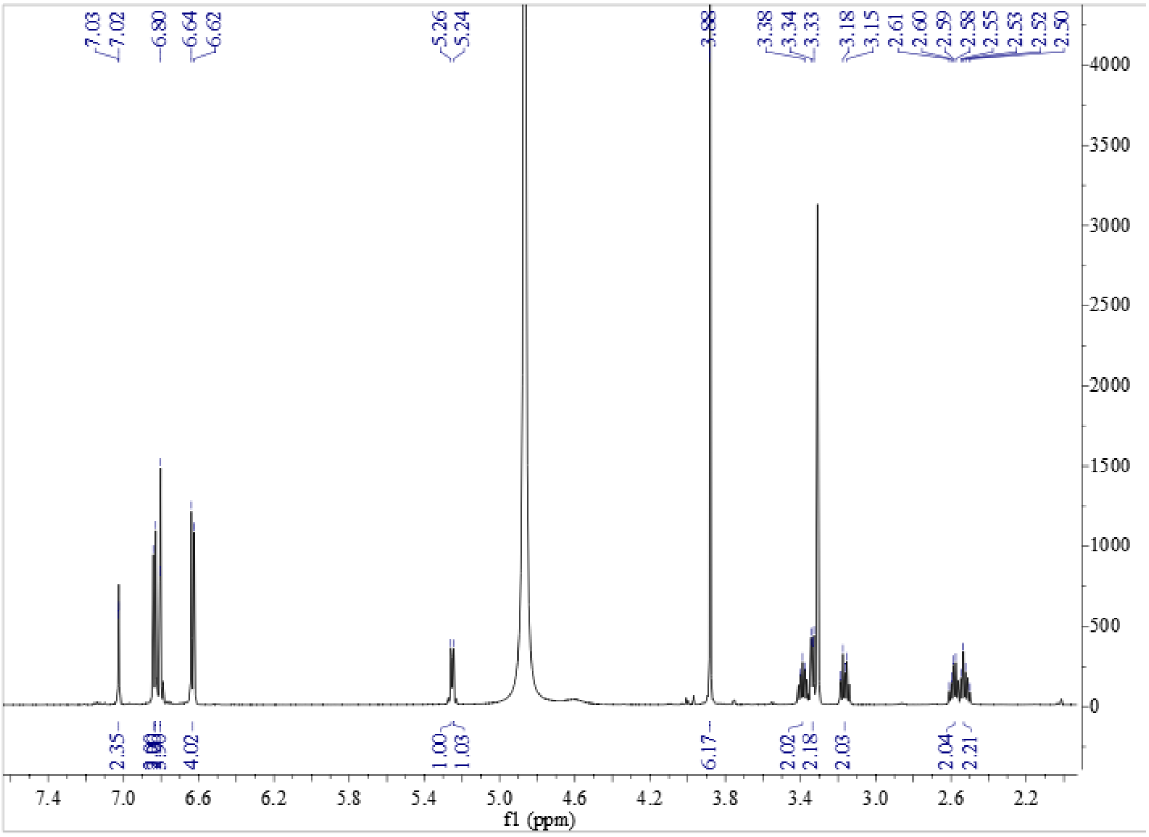

Figure S1-1. ^{1}H NMR spectrum (600 MHz) of compound **1** in methanol-d_4

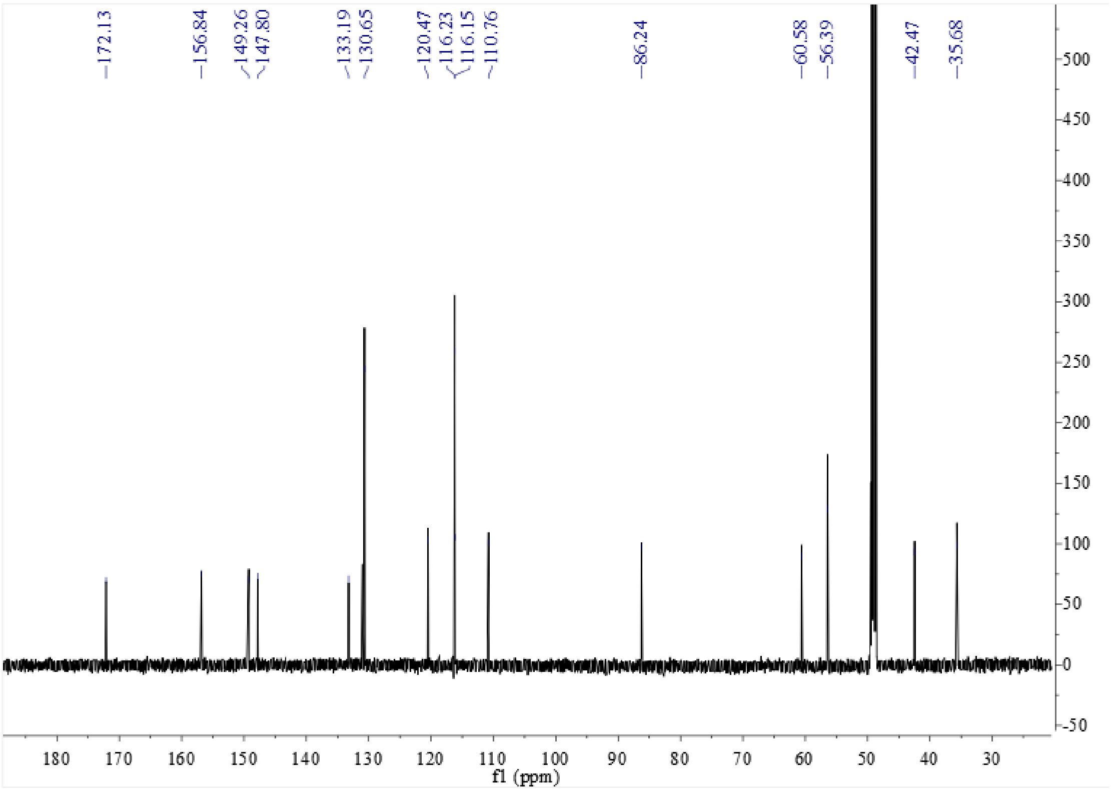

Figure S1-2. ^{13}C NMR spectrum (150 MHz) of compound **1** in methanol-d_4

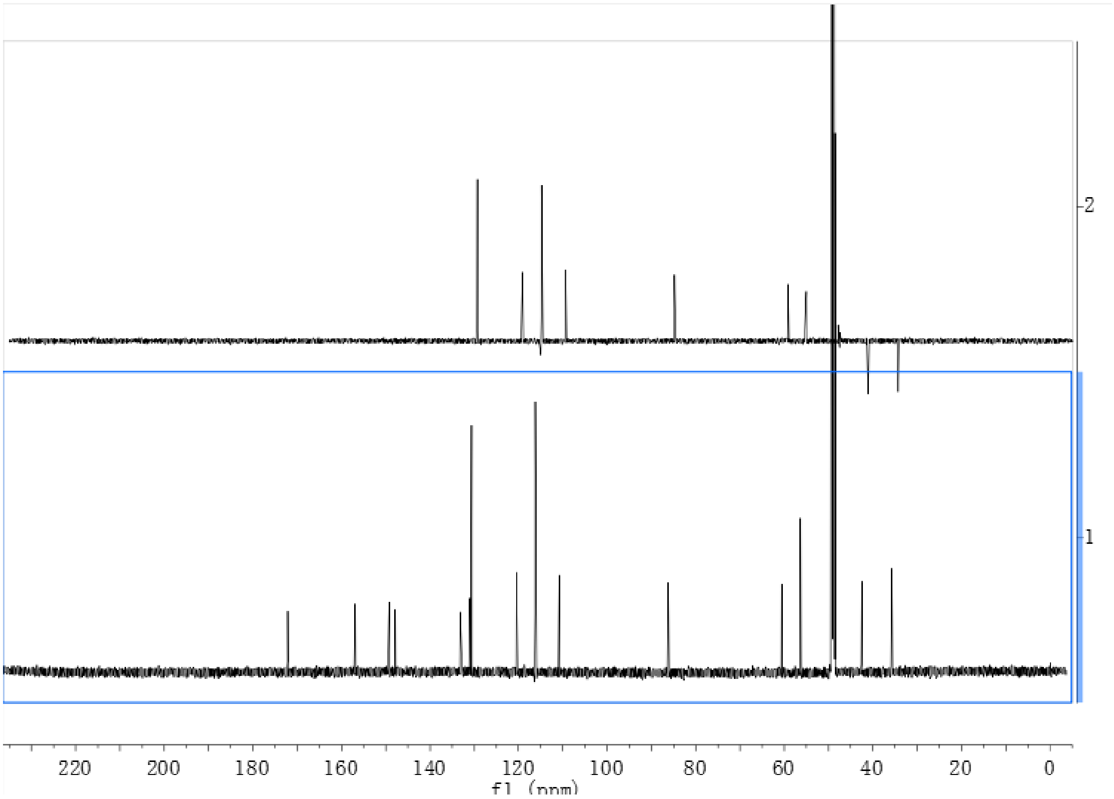

Figure S1-3. DEPT-135 spectrum (150 MHz) of compound **1** in methanol-d_4

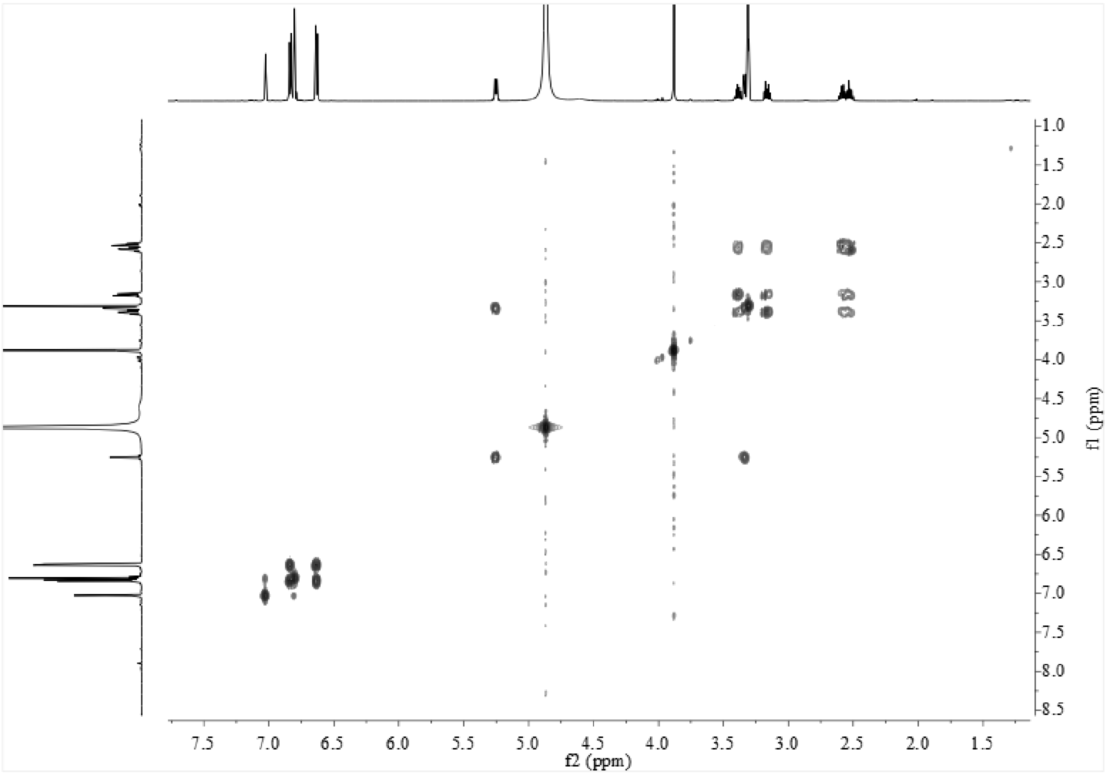

Figure S1-4. ^{1}H-^{1}H COSY spectrum (600 MHz) of compound **1** in methanol-d_4

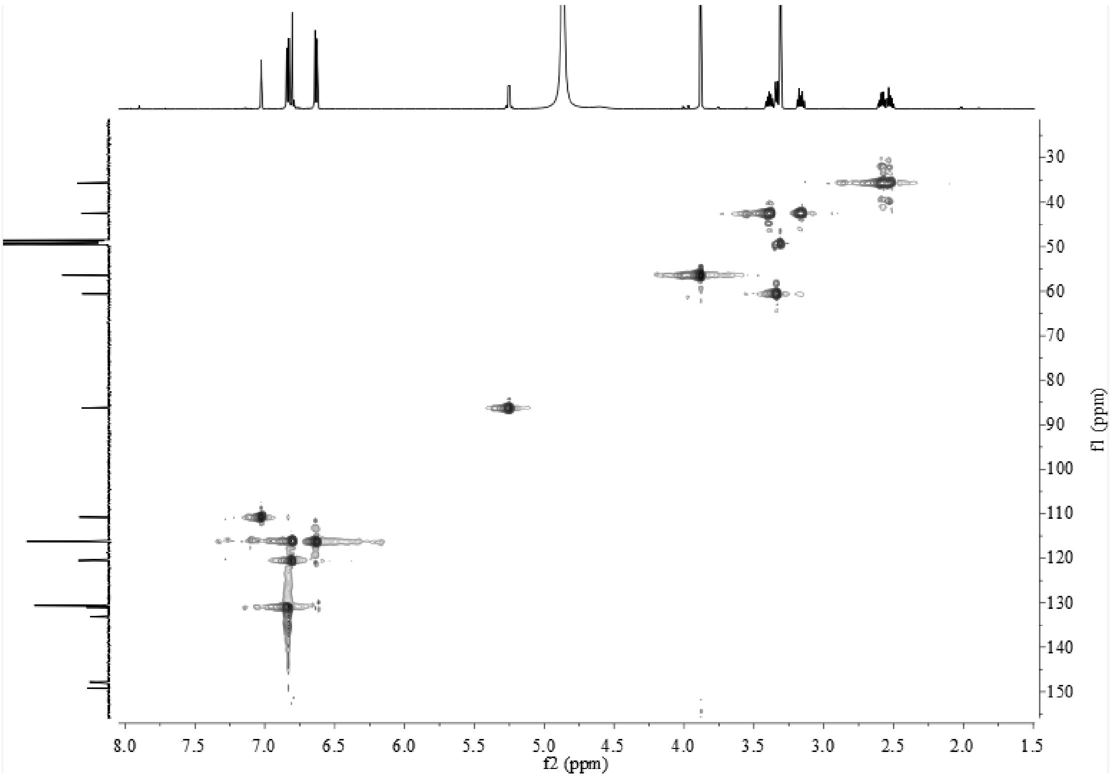

Figure S1-5. HMQC spectrum (150 MHz) of compound **1** in methanol-d_4

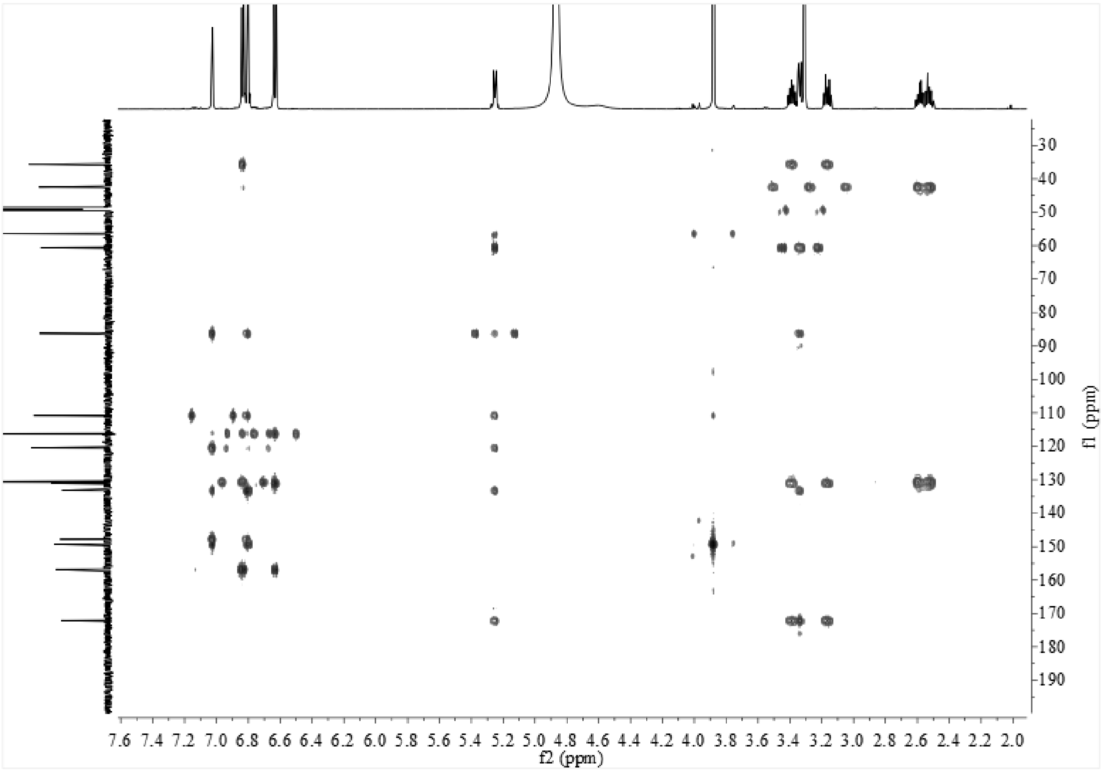

Figure S1-6. HMBC spectrum (150 MHz) of compound **1** in methanol-d_4

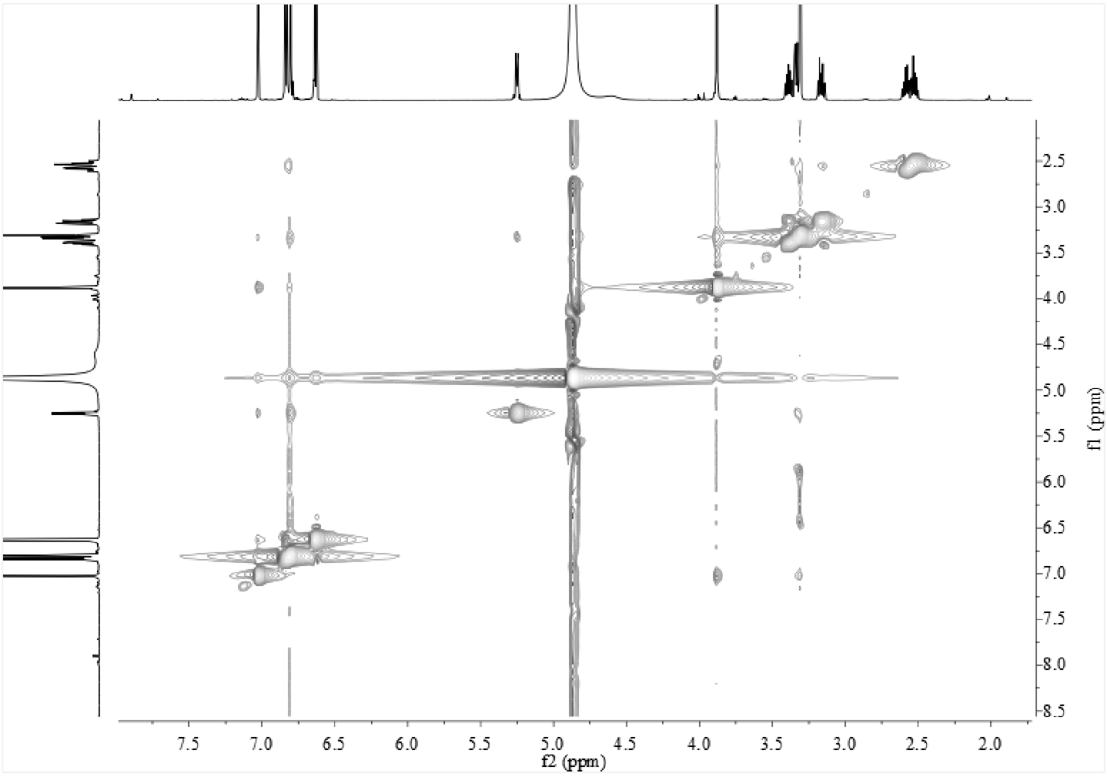

Figure S1-7. NOESY spectrum (600 MHz) of compound **1** in methanol-d_4

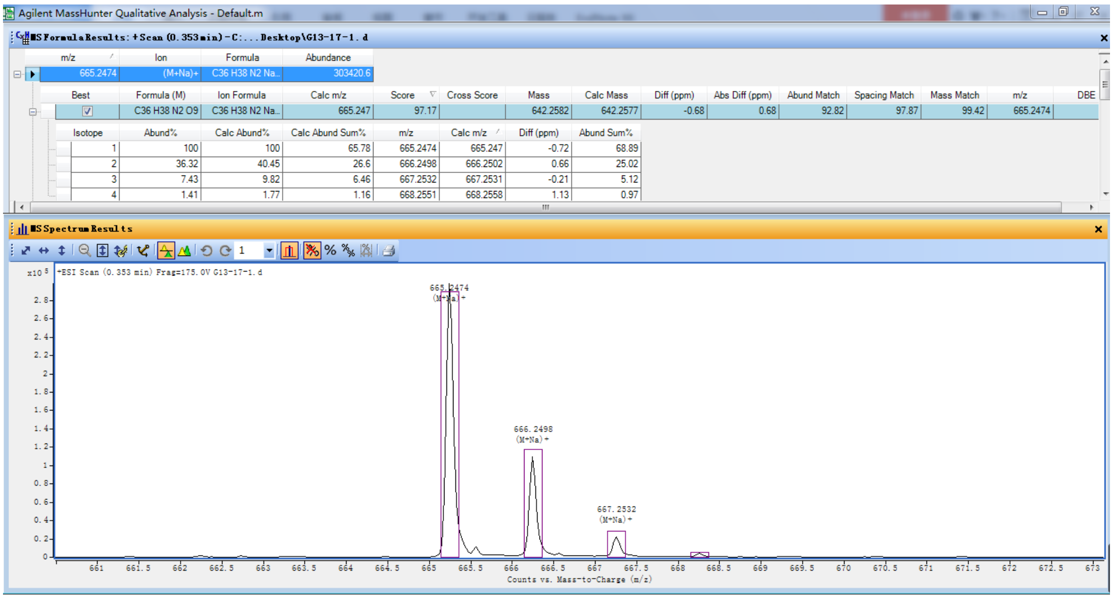

Figure S1-8. HR-ESI-MS of compound **1**

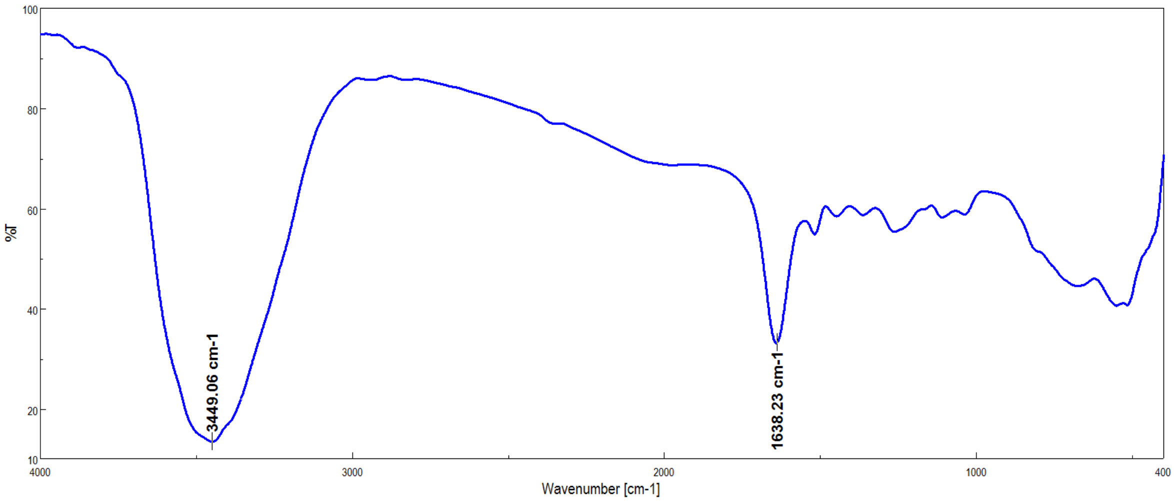

Figure S1-9. IR spectrum of compound **1**

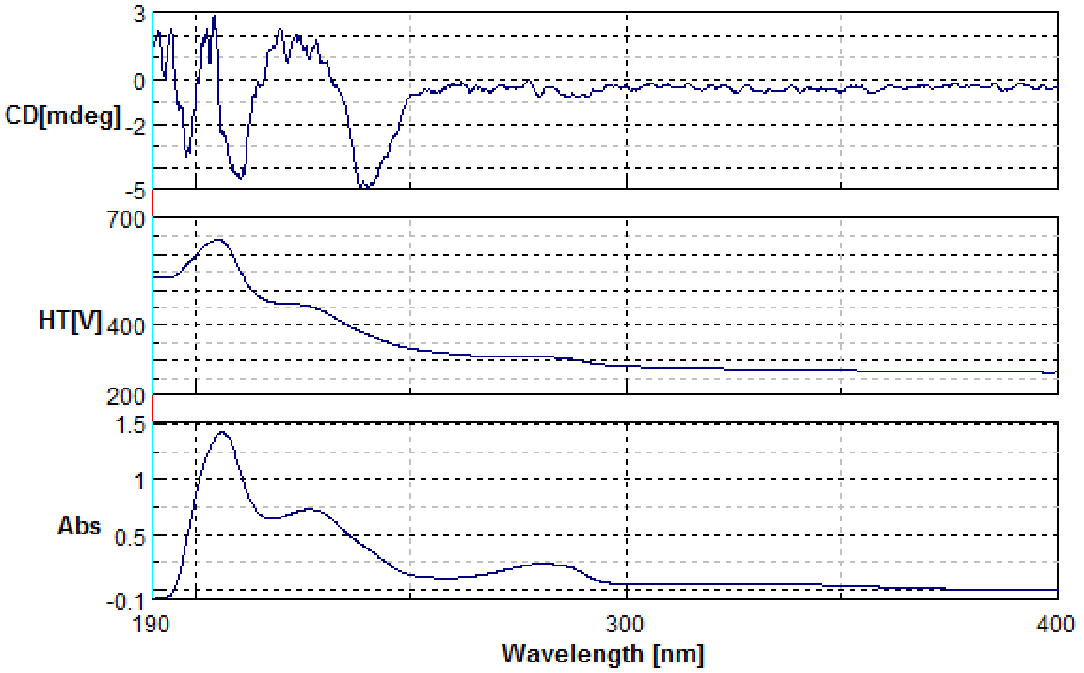

Figure S1-10. CD spectrum and UV spectrum of compound **1** in MeOH

Label	Conformer	Boltzmann weighting factors
C1	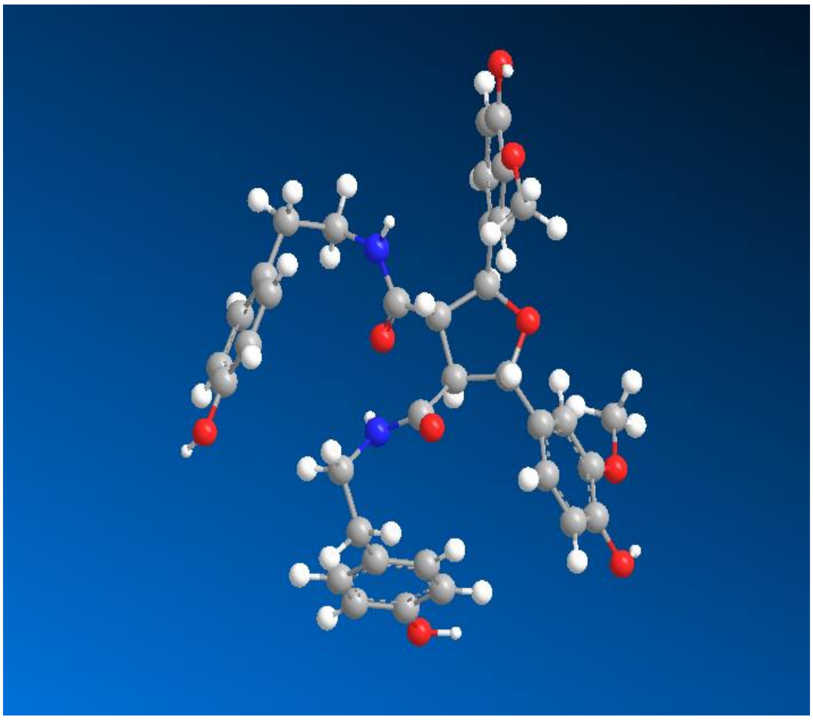	99.81%

Figure S1-11. B3LYP/6-31 G optimized lowest energy 3D conformer of **1**.

Comound	conformer	ΔE (kcal/mol)	Population (%)
**1**	C1	0	99.81
	C2	0.0066495	0.09
	C3	0.0066496	0.09
	C4	0.0080866	0.02

[a]Relative to conformer C1 with E6-31+G(d) = -2181.8637403 Kcal/mol. [b]Calculated using free energy values from Gaussian 03W according to ΔG = -RT In K.

Table S1. B3LYP-Calculated relative energies (kcal mol^{-1}) and conformational population (%) for the most stable conformers of (7*S*,7'*S*,8*S*,8'*S*)-**1**.

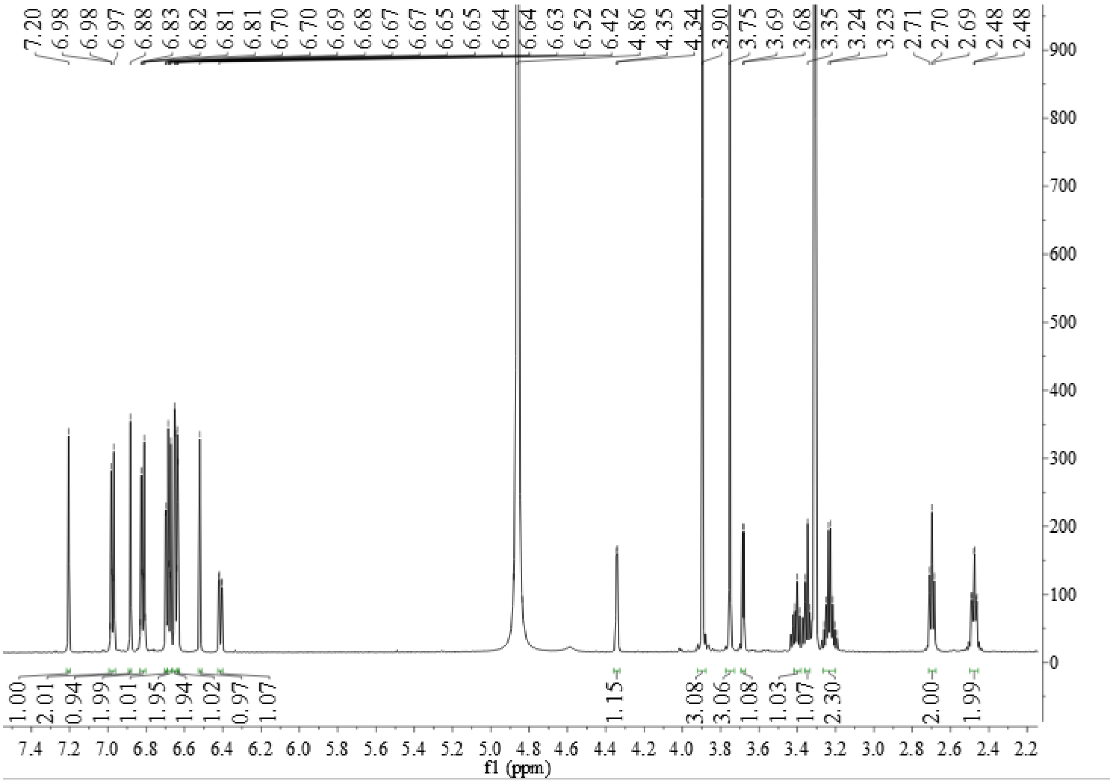

Figure S2-1. ^{1}H NMR spectrum (600 MHz) of compound **2** in methanol-d_4

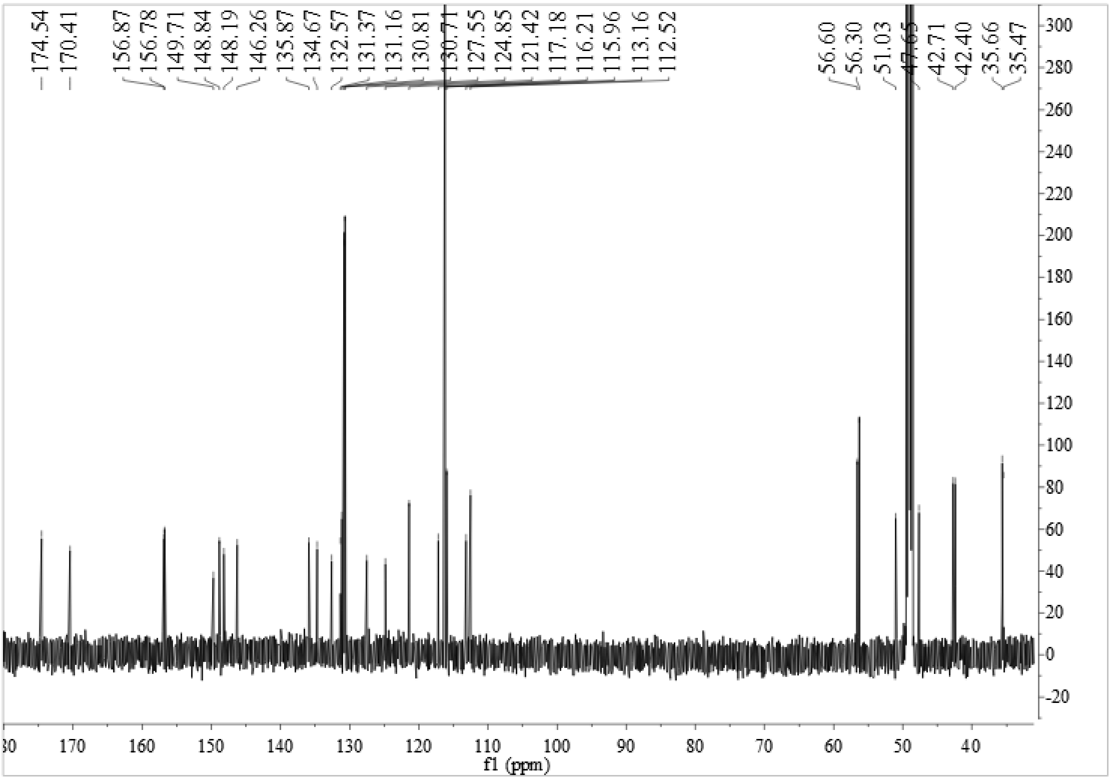

Figure S2-2. ^{13}C NMR spectrum (150 MHz) of compound **2** in methanol-d_4

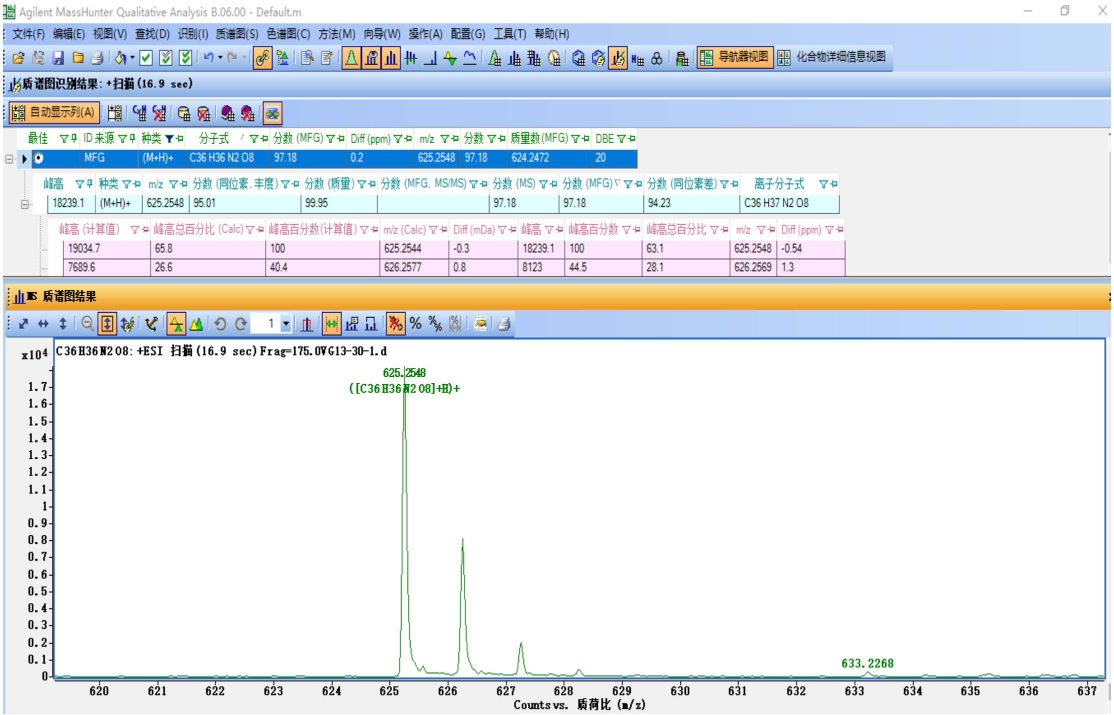

Figure S2-3. HR-ESI-MS of compound **2**

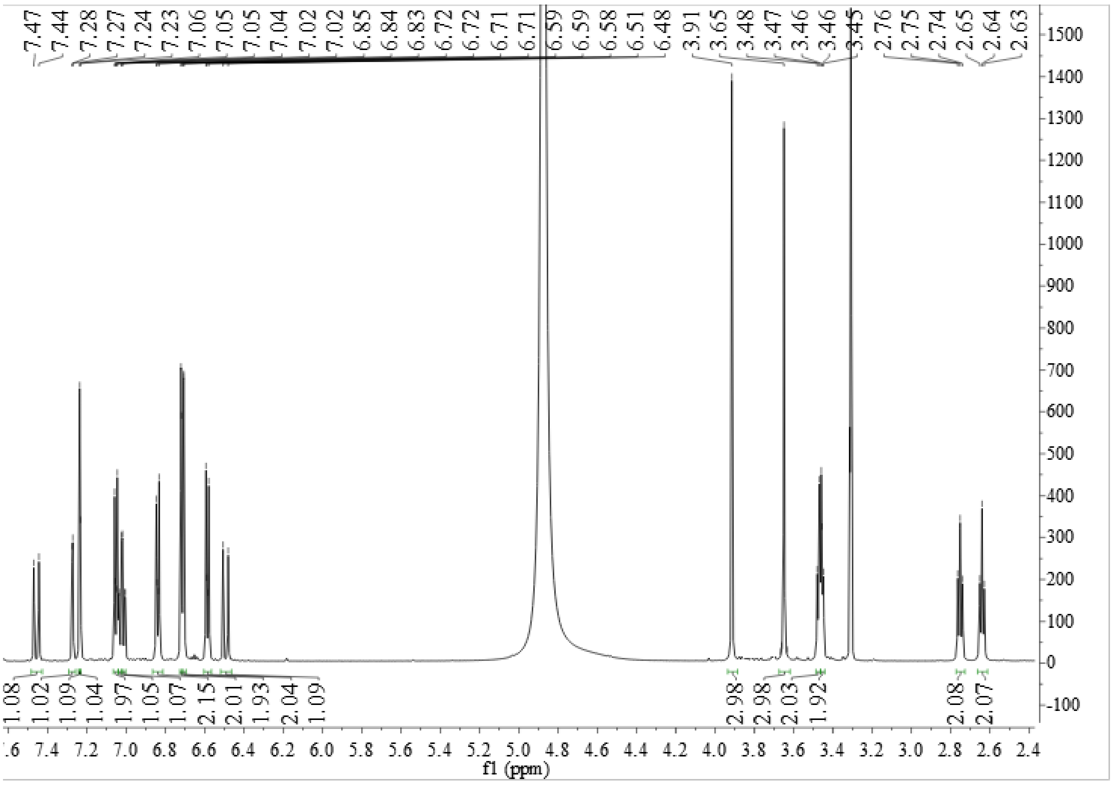

Figure S3-1. ^{1}H NMR spectrum (600 MHz) of compound **3** in methanol-d_4

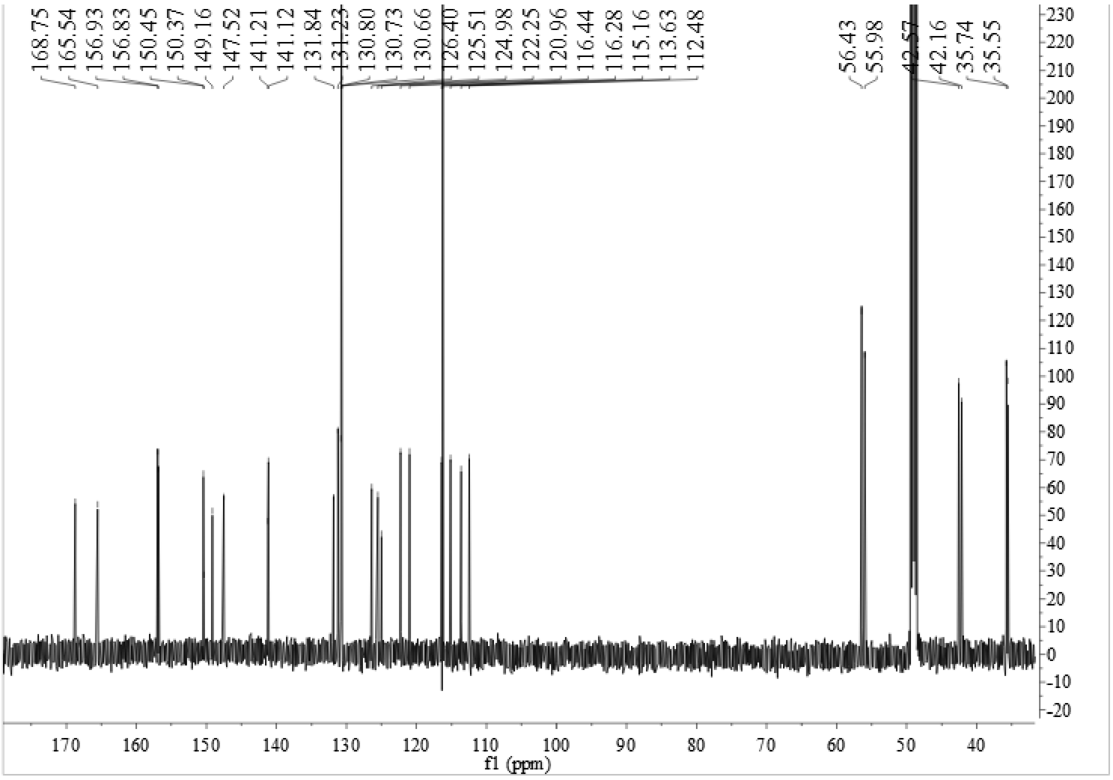

Figure S3-2. ^{13}C NMR spectrum (150 MHz) of compound **3** in methanol-d_4

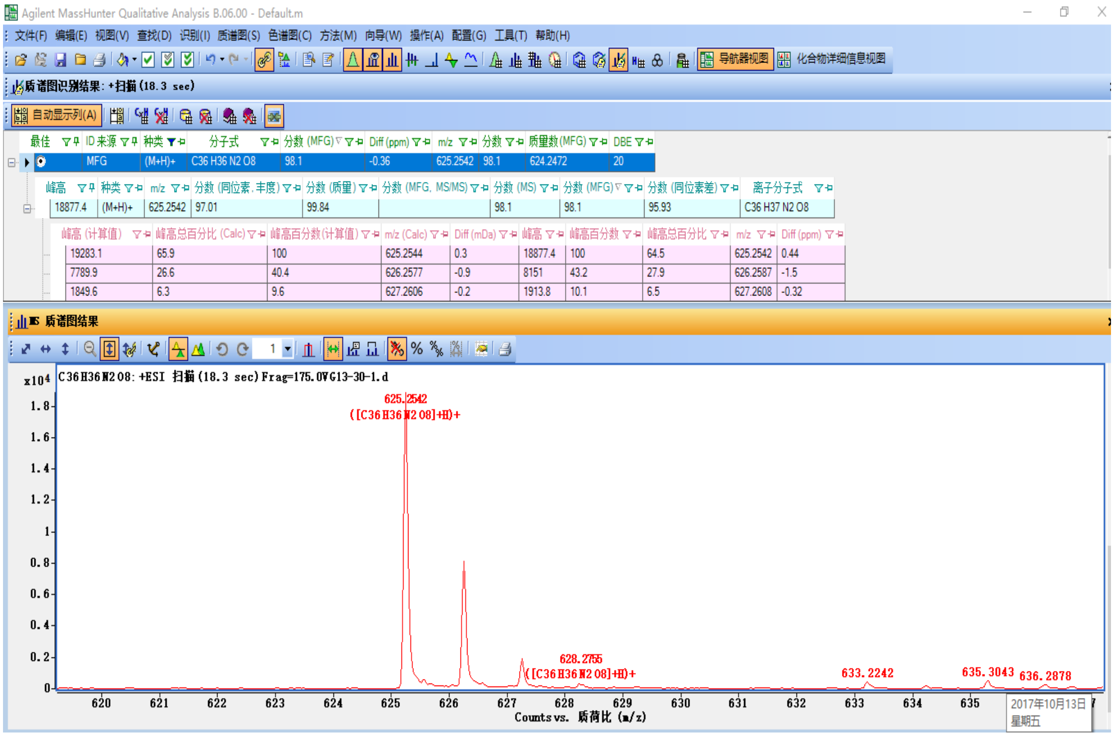

Figure S3-3. HR-ESI-MS of compound **3**

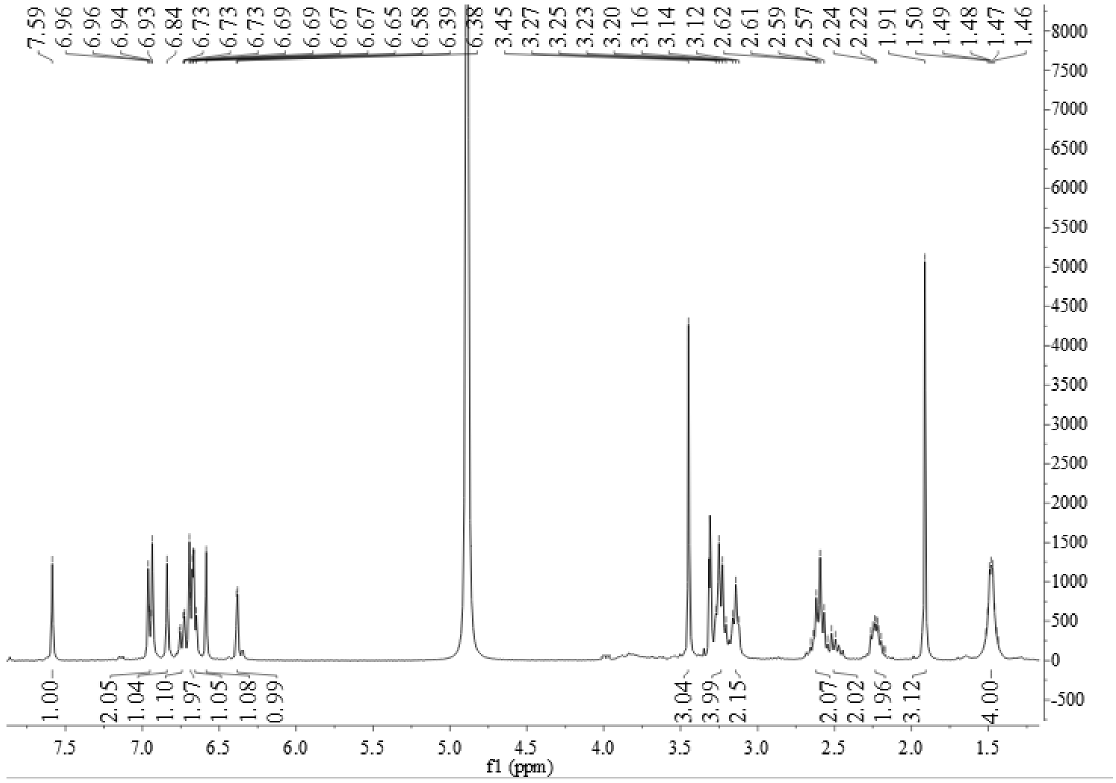

Figure S4-1. ^{1}H NMR spectrum (300 MHz) of compound **4** in methanol-d_4

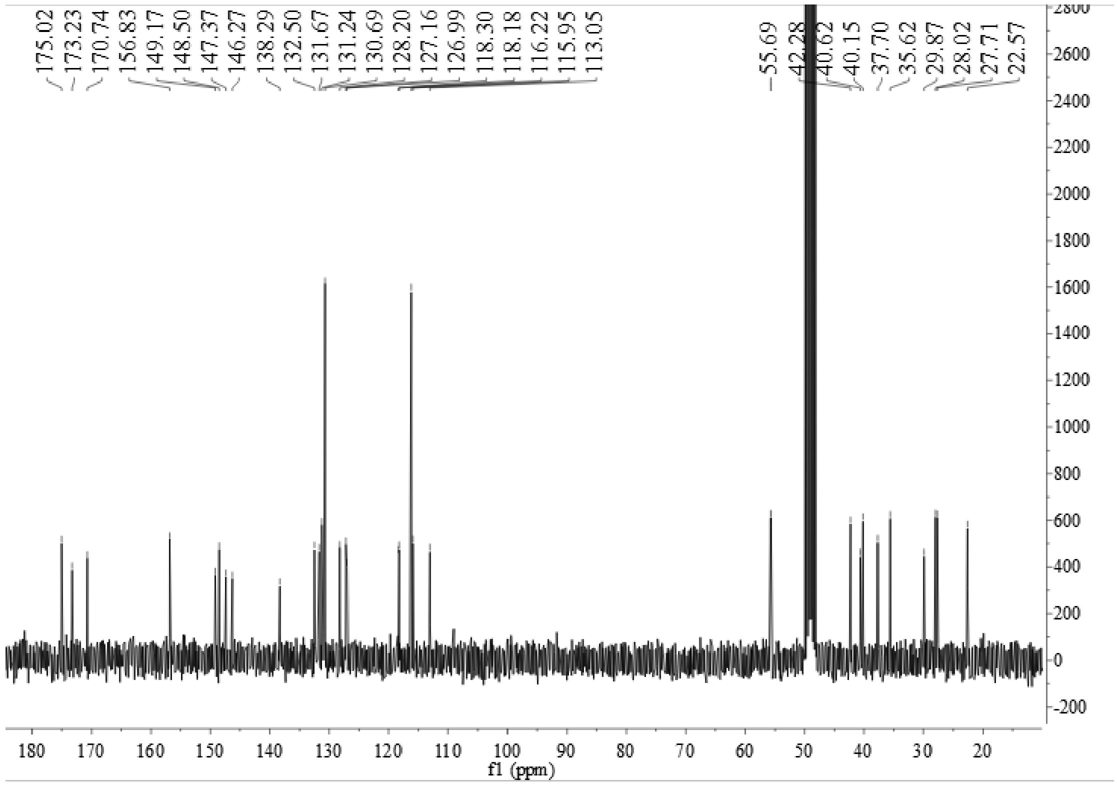

Figure S4-2. ^{13}C NMR spectrum (75 MHz) of compound **4** in methanol-d_4

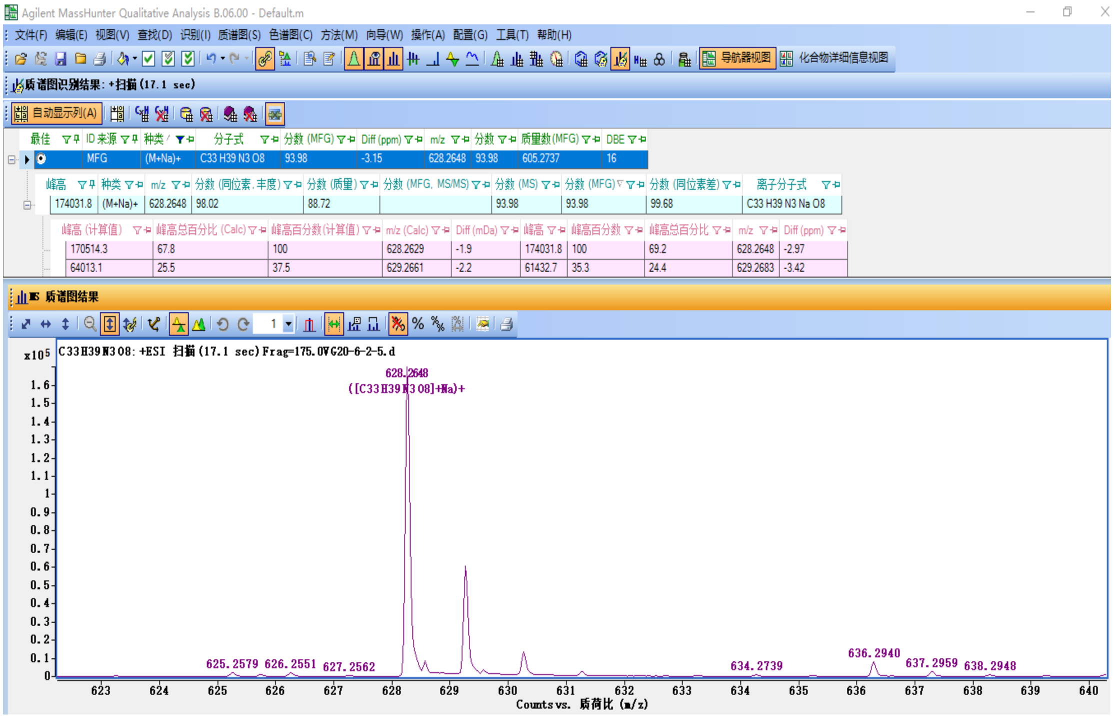

Figure S4-3. HR-ESI-MS of compound **4**

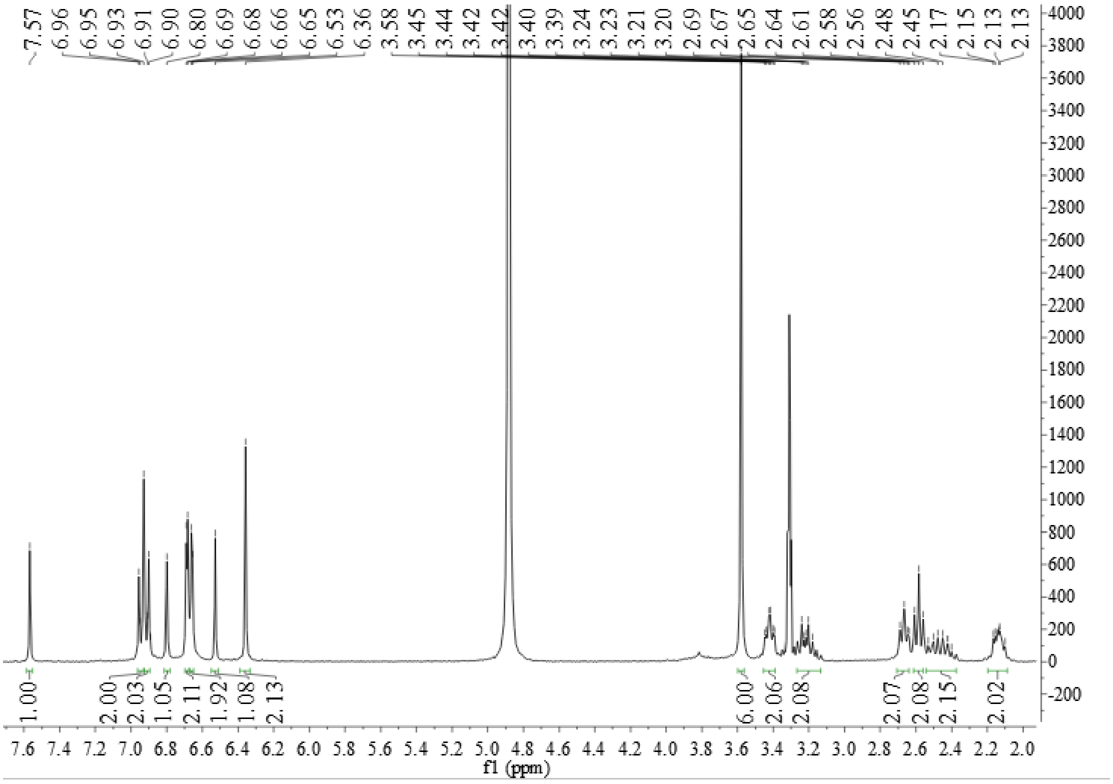

Figure S5-1. ^{1}H NMR spectrum (300 MHz) of compound **5** in methanol-d_4

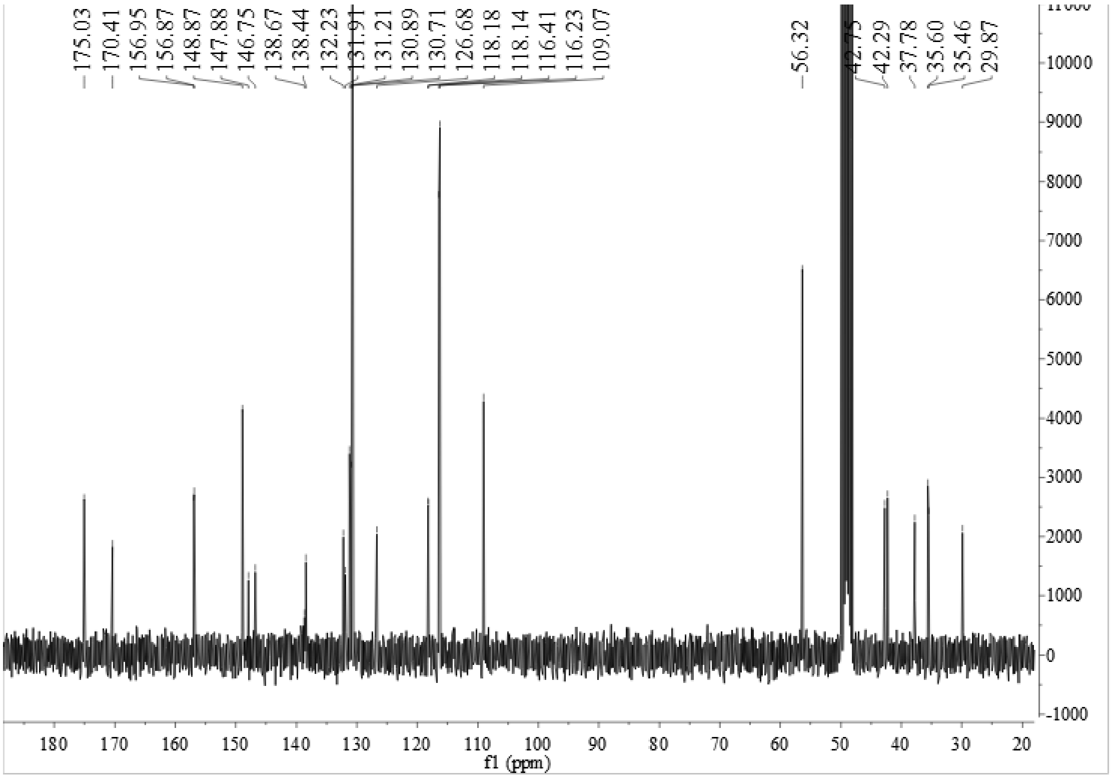

Figure S5-2. ^{13}C NMR spectrum (75 MHz) of compound **5** in methanol-d_4

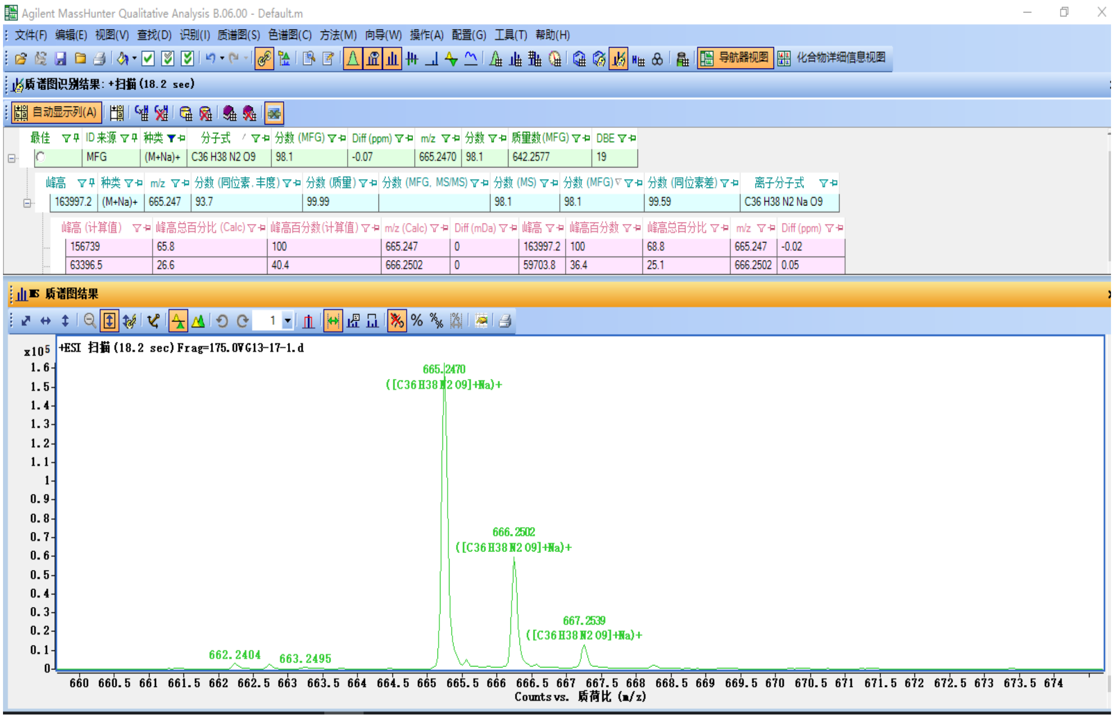

Figure S5-3. HR-ESI-MS of compound **5**
